# Ureterovaginal Fistula Post Vaginal Hysterectomy

**DOI:** 10.7759/cureus.30694

**Published:** 2022-10-26

**Authors:** SaiNidhi G Reddy, Pratap Parihar, Rajasbala Dhande, Roohi G Gupta, Vadlamudi Nagendra

**Affiliations:** 1 Radiodiagnosis, Jawaharlal Nehru Medical College, Datta Meghe Institute of Medical Sciences, Wardha, IND

**Keywords:** excretoryphase, hydronephrosis, fibroid, fistula, ct urography

## Abstract

Middle-aged women with ureterovaginal fistula (UVF) after hysterectomy represent a painful condition for the patients in the community. Accurate diagnosis and proper planning before surgery are essential for effective outcomes. CT urography is the modality of choice in diagnosing ureterovaginal fistula. CT urography helps in evaluating the fistula as well the associated renal complications following the condition. Here we present a case of ureterovaginal fistula reported with a history of vaginal hysterectomy for subserosal fibroid in December 2021.

## Introduction

Since a urogenital fistula forms after gynecological conditions or obstetric surgery, treating urinary incontinence is a difficult undertaking. It is a terrible condition, and clinical problems are made worse by social and psychological elements. Vesicovaginal fistula is the most common urogenital fistula. The ureteral fistula often joins the vagina but sporadically may also connect to the uterus or fallopian tube [1]. Endometriosis, obesity, pelvic inflammatory disease, radiation therapy, and malignant illness of the pelvis are some conditions that increase the risk of developing ureteral fistulas [2]. The symptoms of ureteric damage can manifest either suddenly or more gradually, depending on the type of injury, and they are particularly difficult to detect during laparoscopic procedures. The incidence of ureterovaginal fistula (UVF) has been increasing due to the growing use of the laparoscopic surgical technique [3].

## Case presentation

**Figure 1:**
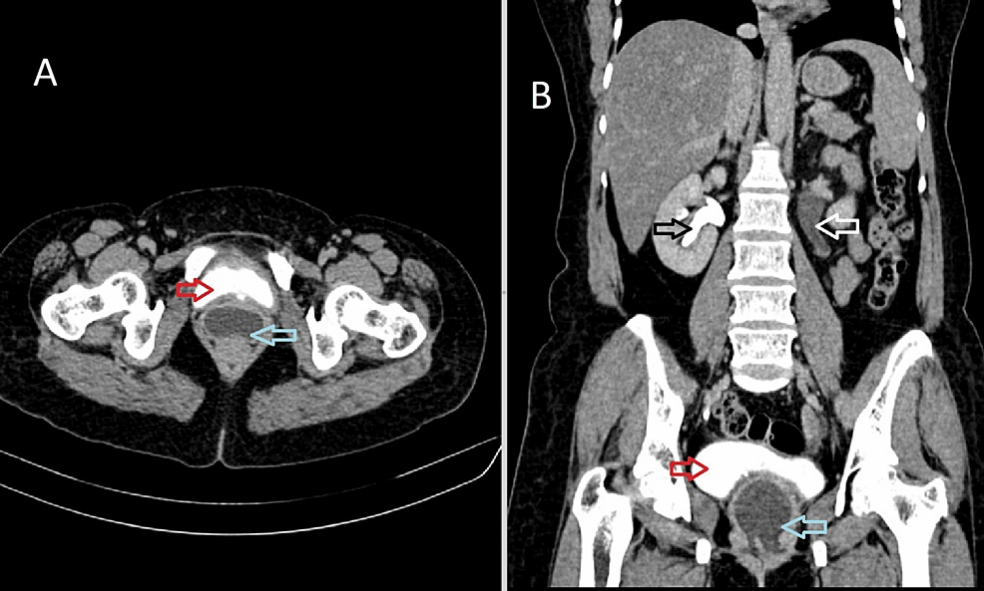
Axial (A) and coronal (B) CT urography excretory phase (3 mins), showing normal excretion of contrast in the right renal collecting system (black arrow) filling the bladder (red arrow), no excretion of contrast in the left renal collecting system (white arrow). The cavity in the vaginal stump appears distended with fluid-filled collection within, possibly urine (blue arrow). CT: Computed tomography

**Figure 2:**
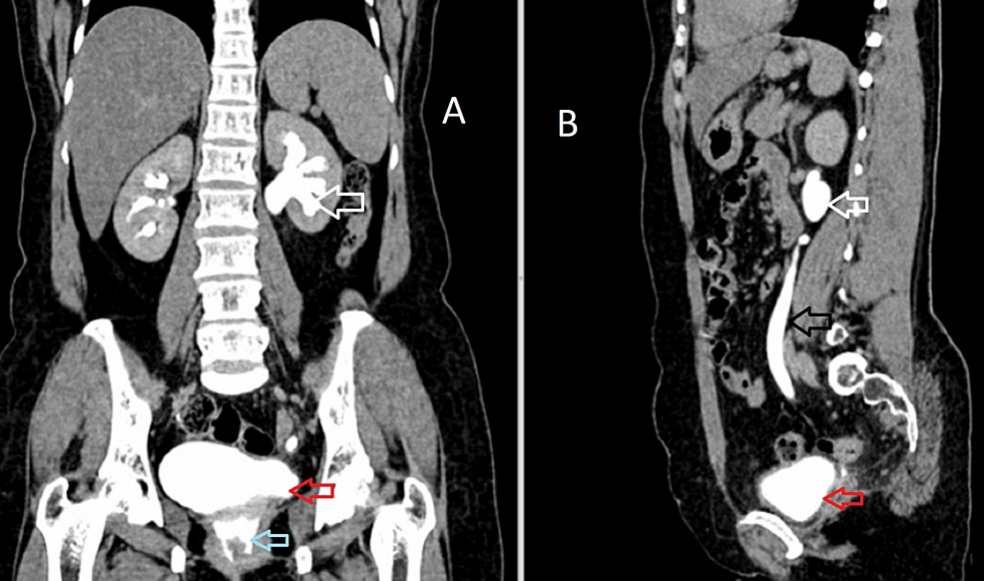
Coronal (A) and sagittal (B) images of the excretory phase (15 mins) shows dilated left pelvicalyceal system (white arrow) and ureter (black arrow) with contrast excretion into the bladder (red arrow). The cavity of the vaginal stump shows e/o excretion of contrast (blue arrow), within suggesting an abnormal connection of the left ureter with the vaginal stump.

**Figure 3:**
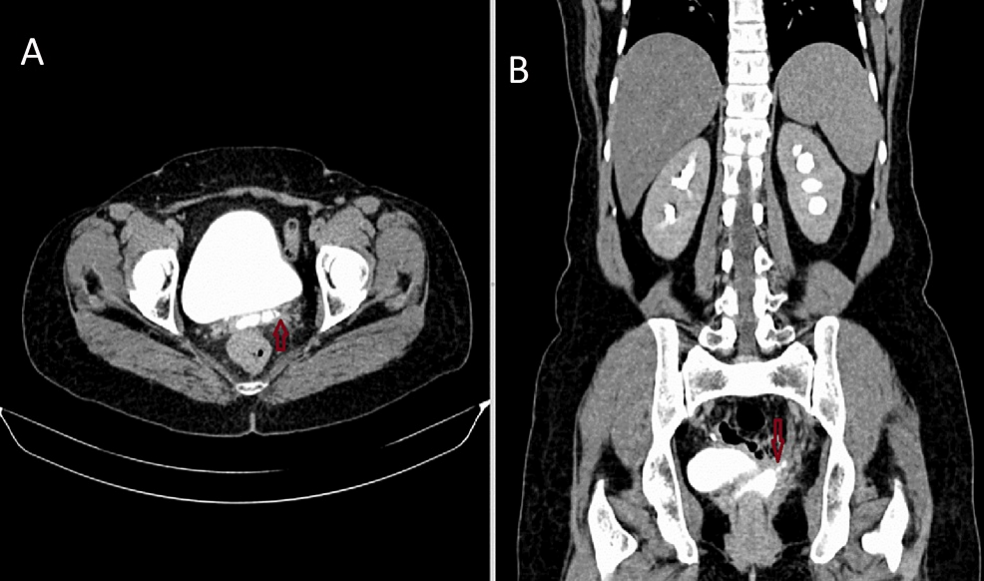
Axial (A) and coronal (B) excretory phase (15 mins) images showing a contrast-filled tract (red arrow) connecting the left ureter to the vaginal stump, confirming ureterovaginal fistula.

A 42-year-old female presented to the department of obstetrics and gynecology with complaints of leaking urine from the vagina for three weeks. On per-speculum examination, there was evidence of dripping urine from the vagina. The patient has a history of large subserosal fibroid for which she was advised for a total hysterectomy. A large subserosal fibroid was identified arising from the posterior wall of the uterus, and a vaginal hysterectomy was performed, leaving the bilateral adnexa and vagina in situ. The procedure was uneventful, and the patient was discharged in three days. The patient developed urinary incontinence and left-sided flank pain two months after the surgery. The patient was then advised for CT urography for further evaluation. On the excretory phase (3 mins), axial and coronal images are showing normal excretion of contrast in the right renal collecting system filling the bladder but no excretion of contrast in the left renal collecting system. The cavity in the vaginal stump appears distended with fluid-filled collection within, possibly urine (Figure 1). Excretory phase (15 mins) axial, coronal, and sagittal images show dilated left pelvicalyceal system and ureter with contrast excretion. The cavity of the vaginal stump shows contrast within, suggesting an abnormal connection of the left ureter with the vaginal stump (Figure 2). We can also appreciate a contrast-filled tract connecting the left ureter to the vaginal stump, confirming ureterovaginal fistula in the axial and coronal excretory phase (15 mins) (Figure 3).

## Discussion

Fistulous tracts between the vagina and ureter are rare, occurring primarily as complications of obstetric and gynaecologic procedures such as low transverse cesarean section or uterine curettage for elective abortion [1]. Pelvic adhesions due to repeated surgery significantly enlarged uterus during abdominal surgery and heavy bleeding that obscures the surgical site during surgery were the main causes of ureteral injury [4]. Clamp, suture ligation, partial or total primary transection, angulation, avulsion, or ischemic necrosis following electrocoagulation are examples of mechanisms that can cause ureter damage during surgical procedures [5]. Endometriosis, obesity, pelvic inflammatory disease, radiation therapy, and pelvic cancer are risk factors for the ureterovaginal fistula [2].

Ureterovaginal fistulas can also be a side effect of pelvic procedures, most commonly hysterectomy. In a 15-year review of genitourinary fistulas at the Mayo Clinic, ureterovaginal fistulas accounted for 31 of 303 cases, with the vast majority resulting from hysterectomy [6]. Due to its potential to result in incontinence, infection, and renal failure, the ureterovaginal fistula is the most dangerous of the urovaginal fistulas [7].

Despite the regular act of micturition, the primary ureterovaginal fistula presentation is an incontinence disease. Incontinence usually develops 1 to 4 weeks after surgery [8]. Affected women will present with urinary outflow from the vagina, and pelvic examination can demonstrate urine leakage at the cervical os [9]. 

Prevention of renal impairment requires early diagnosis. The diagnosis is made using various imaging techniques, which include excretory urography, intravenous CT urography, and magnetic resonance imaging (MRI). Vesicovaginal fistula frequently coexists. The diagnosis is confirmed by the evidence of contrast extravasation from the ureter into the vagina, which may be seen on a delayed phase CT scan. Patients with renal failure or conditions where CT is contraindicated can use MRI [10]. MRI can be performed using contrast-enhanced T1-weighted image sequences or non-contrast T2-weighted image sequences. In situations involving renal insufficiency, T2-weighted MR urography is employed [11].

The fistula may be able to heal with conservative therapy using a percutaneous nephrostomy tube and/or ureteral stent. Once the ureterovaginal fistula is diagnosed, an immediate ureteral stent or nephrostomy tube placement should be tried. Prompt urine drainage is necessary to maintain renal function even if surgery is subsequently required [12,13].

## Conclusions

The morbidity linked to ureteral injury may be severe, leading to a protracted hospital stay, a poor surgical outcome, additional invasive procedures, decreased renal function, and a lower quality of life for the patient. Following a vaginal hysterectomy, the risk of ureteral complications is similar to that of an open laparotomy. The most effective ways to avoid harm are surgical skills, understanding of the anatomy of the pelvic wall, and meticulous identification and mobilization of the ureter. It's essential to get a diagnosis early to avoid needing long-term care. In the vast majority of patients with early diagnosis, endoscopic procedures are adequate. Our case was managed with a percutaneous nephrostomy tube followed by antegrade stent placement. However, severe ureteral strictures, urogenital fistulas, and renal insufficiency caused by ureteral injuries necessitate more comprehensive reconstructive surgery.
